# Maternal effects and *Symbiodinium* community composition drive differential patterns in juvenile survival in the coral *Acropora tenuis*

**DOI:** 10.1098/rsos.160471

**Published:** 2016-10-19

**Authors:** Kate M. Quigley, Bette L. Willis, Line K. Bay

**Affiliations:** 1 College of Marine and Environmental Sciences, and ARC Centre of Excellence for Coral Reef Studies, James Cook University, Townsville, Queensland 4811, Australia; 2 AIMS@JCU, Australian Institute of Marine Science and James Cook University, Townsville, Queensland 4811, Australia; 3 Australian Institute of Marine Science, PMB 3, Townsville, Queensland 4810, Australia

**Keywords:** *Symbiodinium*, settlement, juvenile, survivorship, maternal effects, coral reef

## Abstract

Coral endosymbionts in the dinoflagellate genus *Symbiodinium* are known to impact host physiology and have led to the evolution of reef-building, but less is known about how symbiotic communities in early life-history stages and their interactions with host parental identity shape the structure of coral communities on reefs. Differentiating the roles of environmental and biological factors driving variation in population demographic processes, particularly larval settlement, early juvenile survival and the onset of symbiosis is key to understanding how coral communities are structured and to predicting how they are likely to respond to climate change. We show that maternal effects (that here include genetic and/or effects related to the maternal environment) can explain nearly 24% of variation in larval settlement success and 5–17% of variation in juvenile survival in an experimental study of the reef-building scleractinian coral, *Acropora tenuis*. After 25 days on the reef, *Symbiodinium* communities associated with juvenile corals differed significantly between high mortality and low mortality families based on estimates of taxonomic richness, composition and relative abundance of taxa. Our results highlight that maternal and familial effects significantly explain variation in juvenile survival and symbiont communities in a broadcast-spawning coral, with *Symbiodinium* type A3 possibly a critical symbiotic partner during this early life stage.

## 1. Introduction

Ecological and biological processes commonly recognized to shape community composition include predation, competition and stochastic events that naturally change the abundance and diversity of species through time. However, for coral reef communities facing an increasing number of pressures that impact their productivity, health and composition [1], processes influencing the replenishment of coral populations must also be considered. For reefs to recover and contend with mounting pressures, larval settlement and juvenile survival must be at least equal to or higher than mortality rates of mature colonies. Studies to date suggest that these early life-history processes may be substantially influenced by parental genotype and condition; however, the roles of parental genetics and transgenerational effects in enhancing larval and juvenile survival have only begun to be empirically tested for corals [2–7]. Quantification of the relative contributions of genetic and environmental impacts on larval settlement and juvenile survival is needed to predict how coral populations are likely to respond to changing selective pressures in the future. Moreover, understanding the potential of pre- and post-recruitment processes to regenerate coral populations and rates of recovery is essential to predict at-risk and sensitive areas unable to compensate for natural and human-induced disturbance [8–10].

Annual coral recruitment rates vary from 35- to 100-fold and are only partly explained by adult colony abundance and fecundity [9]. Consequently, additional factors besides strict stock–recruitment relationships determine juvenile abundance on reefs. Once metamorphosed, many factors influence juvenile abundances and species composition of coral assemblages, including abiotic (e.g. environmental conditions and storms [11,12]) and biotic factors (e.g. parental reproductive mode [8], accidental predation by herbivorous fish [13,14], juvenile growth and intra/inter-species competition [15]). The timely and specific acquisition of different clades/types of *Symbiodinium*, a key dinoflagellate genus found in corals, has also been linked to juvenile survival, with clades A and D found in greater proportions in surviving juveniles of *Acropora yongei* [16]. *Acropora millepora* juveniles also survived better in low-light treatments when symbiont communities were composed of roughly equal proportions of C1 and D *Symbiodinium*, whereas corals with C1-dominated communities survived better in high-light treatments [17]. Although it is clear that environmental factors play a role in coral juvenile survival, further work is needed to elucidate and quantify the contributions of parental genotypes versus non-genetic maternal effects and the acquisition of symbionts on coral juvenile fates.

Maternal effects have been shown to govern variability in a range of traits among individual gametes and larvae [18] and contribute to structuring communities of marine fish and invertebrates [7]. For broadcasting corals that do not invest resources in either mating or parental care and for which gamete production is the sole reproductive investment, it is particularly critical to understand factors influencing gamete number and quality, which in turn, affect several aspects of early life-history stages [19]. For example, maternal provisions of lipid fulfil almost half the metabolic needs of coral larvae [20,21] and have been shown to influence larval growth and survivorship in both fish and corals [22,23]. However, the quality of maternal provisioning is dependent on the environmental conditions experienced by the mother. Variations in larval and egg morphology associated with hormone exposure within the maternal environment [24] and age at maternal reproduction [25] are further demonstrations of how non-genetic maternal effects can impact offspring fitness traits. While these impacts are not due to parental genetics *per se*, they are a direct result of parental physiology impacting later generations.

Parental genotypes can contribute significantly to offspring fitness and their potential for local adaptation through selection for optimal expressions of offspring traits under local environmental conditions. In fish, maternal and paternal genetic factors are known to dictate a range of key larval traits, including size and growth, and to underpin variability in larval survival and settlement, and juvenile survivorship [26]. In corals, the parental identity of larvae influences up to 47% of settlement success in the spawning coral *A. millepora* in aquaria [5]. Recent tank experiments with brooding corals found that 94% of the variability in juvenile survivorship and 27–30% of variability in juvenile growth in aquaria were due to familial genotypes [6]. Parental combinations also impact other early life-history traits in corals, such as protein content, affinity to settlement cues, fertilization success and larval heat tolerance [2,4,27]. Lipid profile characteristics are also under substantial parental genetic regulation in human studies [28,29]. Therefore, quantifying the impacts of parental genotype on processes governing recruitment success is key to understanding local adaptation and the potential for change; however, the impacts of parental genotype on a range of early life-history processes in corals, notably juvenile survivorship in the wild, are still unknown.

To further current understanding of the extent to which parental identities drive variation in larval survival, larval settlement and juvenile survival, this study quantified parental effects on key early life-history stages in the broadcasting coral *Acropora tenuis.* Using larvae from intrapopulation and interpopulation crosses, we determine the impacts of parental identity on larval survival, weight and settlement, and on juvenile survival in the field. We also compare *Symbiodinium* communities among families with high and low juvenile mortality and discuss potential pathways that are likely to underpin the patterns found.

## 2. Material and methods

### 2.1. Collection of colonies

For interpopulation and intrapopulation crosses, 14 reproductively mature colonies of *Acropora tenuis* were collected at the end of October 2013 from Wilkie Reef (13°46′44.544′′ S, 143°38′26.0154′′ E) in Princess Charlotte Bay in the far northern sector of the Great Barrier Reef (GBR). Corals were transported by boat to Orpheus Island Research Station (OIRS) and acclimated in outdoor holding tanks for 22 days under constant flow-through conditions. Ten colonies of *A. tenuis* were collected from South Orpheus on 19 and 20 November 2013 (18°39′49.62′′ S, 146°29′47.26′′ E) and housed at OIRS under the same flow-through conditions for 1–2 days prior to spawning. Colonies collected were separated by at least 5 m, and given differences in colony colour and limited success of asexual reproduction in corymbose corals, they were assumed to represent distinct genotypes.

### 2.2. Reproductive crosses and experimental design

Following signs of spawning imminence in *A. tenuis*, i.e. the appearance of egg–sperm bundles under the oral disc and polyp setting behaviour (polyps extended but tentacles retracted), individual colonies were isolated until gametes were released between 17.30 and 19.30 on 21 November. Gamete bundles were collected for crosses among four Orpheus Island (O) and four Wilkie (W) colonies. Gametes were gently skimmed from the surface of the water, then eggs and sperm were separated by washing through a 1 µM sieve three times. The density of sperm was quantified with a Neubauer haemocytometer in triplicate replicates, and sperm mixtures then diluted to a density of approximately 1 × 10^6^ sperm per litre to optimize fertilization success [30].

Twenty-five distinct coral families were produced by adding equal numbers of eggs from one parental colony to equal concentrations of sperm from a second parental colony and allowing eggs to fertilize for 3 h (electronic supplementary material, S1). Aliquots of developing embryos were taken from each family every hour for 3 h to quantify fertilization success and cell division to the four-cell stage [31]. Once a majority of the fertilized eggs of all families had reached the four-cell division stage, the number of zygotes was counted in four replicate 0.5 ml samples with a light microscope in order to stock culture replicates with equal densities of developing embryos. Three culture replicates per cross were established at a density of 1.5 four-celled embryos per 2 ml (approx. 20 475 embryos per family summed across the three replicates).

### 2.3. Larval rearing, settlement and field deployment

Each family was cultured in triplicate 9.1 l plastic containers in a temperature-controlled room set at 25°C. Two culture replicates received a constant flow of 26°C filtered seawater (FSW), while the third replicate was static. To maximize the chance of juveniles surviving, we chose to use both static and flow-through larval cultures because of variability in the success of both methods. FSW was drawn off the reef crest at OIRS and stepped through a filter system consisting of 50, 25, 10 µM filters followed by a final UV treatment. Each flow-through replicate culture had an outflow covered by a 10 µM filter and air curtain to prevent eggs and larvae from collecting on the outflow filter. Complete filtered FSW changes were undertaken on all cultures the first day post-fertilization (pf) and on days 3, 5, 8, 10. By days 3 and 4 pf, larvae were ciliated and motile, consistent with the 96 h stage of larval development reported for this species [31]. All culture replicates for Families 25 and 29 perished, despite culturing conditions identical to those of other families. Families were scored as alive or dead, in the former case (alive) based on the survival of some larvae across all three culture replicates.

Once settlement trials confirmed competent settlement behaviour [32] on the sixth-day post-fertilization, flow-through was turned off and a crustose coralline algae (CCA) settlement cue was provided to larvae. Chips of freshly collected CCA (*Porolithon onkodes*) from SE Pelorus (18°33′34.87′′ S, 146°30′4.87′′ E) were placed onto: (i) glass slides in culture replicate one, (ii) calcium carbonate plugs positioned in a plastic stand in culture replicate two or (iii) directly onto the bottom of culture replicate vessel three. Settlement was quantified as counts of settled juveniles per culture replicate on days 9, 11, 14 and 17 pf, and then summed. Therefore, settlement was quantified as the total number of settled juveniles 17 pf per family per replicate. Metamorphosed individuals (juveniles) were out-planted 19 days pf to Little Pioneer Bay, Orpheus Island (18°36′06.2′′ S, 146°29′19.1′′ E). Glass sides and calcium carbonate plugs with attached juveniles were placed in custom-built holders attached to star pickets hammered into the reef substratum. Because juveniles settled onto culture replicate 3, these vessels were also placed in the field. These settlement containers from culture replicate 3 for each family were inverted and strung randomly on horizontal metal rods attached to star pickets. Rods were arranged so that containers were located equidistantly above coral rubble. Plastic mesh netting was affixed to the opening of each container using rubber bands to enable water exchange while preventing predation. In addition, the small plastic stand used to hold the calcium carbonate plugs in culture replicate 2 had high numbers of settled juveniles; therefore, these plastic stands were also attached inside the containers from culture replicate 3 corresponding to their respective family. Juveniles were left in the field for 25 days, after which time the number of surviving juveniles in each family was quantified. All juveniles were subsequently preserved in 100% ethanol.

### 2.4. Larval weights

Approximately 20 swimming larvae per family were fixed in 100% ethanol 18 days pf and stored at −20°C. Two larvae per family were freeze-dried overnight and then individually weighed to the nearest 0.0001 mg on a UMX2 ultra microbalanace (Mettler Toledo; Greifensee, Switzerland). Larval weights were combined across families to enable statistical inferences at the level of dam (*n* = 4–8 larvae/dam) or population cross (*n* = 10–13 larvae/population cross). Larvae could not be collected from the two families in which all larvae died (F_25_ and F_29_). Larvae of a third family (F_23_) were inadequately preserved for larval weight analysis. Although collecting larvae 12 days after settling had commenced may have biased calculations of mean larval weights, if larvae from a specific weight class were to settle first and become unavailable for collection, this issue would have been common to all families. Therefore, any potential bias in calculating mean larval dry weight per family because of sampling time would not have affected comparisons among families.

### 2.5. Statistical design and analysis

Statistical tests were undertaken to evaluate parental effects on larval survivorship, larval weight, settlement success and juvenile survivorship. Data were partitioned in four ways: (i) population purebreds versus population hybrids (OO, WW versus OW, WO), (ii) population cross (OO versus WW versus OW versus WO), (iii) family (i.e. parental cross) and (iv) maternal or paternal identity (dam, sire). Assessments of purebreds versus hybrids and the four types of population crosses evaluated if local adaptation at the population level might be driving patterns at either the family or individual parent level. All three culture replicates per family were used to quantify larval survival (larvae surviving/culture). Only two culture replicates were used to quantify settlement because settlement was extremely low in all larval families in the first replicate (i.e. where settlement surfaces were glass slides). All juveniles on the calcium carbonate plugs died due to algal infestation, so only juveniles that had settled on the plastic plug stands were used from culture replicate 2. Because juveniles that had settled on the plug stands in replicate 2 and on plastic containers in replicate 3 were physically combined in the field, they were summed to quantify juvenile survival, which was measured as the number of juveniles surviving per family. All statistical tests were run in R [33], with an alpha level at 0.05. Means are reported plus/minus their standard errors (±s.e.).

#### 2.5.1. Population effects on larval survival, weight and settlement, and on juvenile mortality

Two-sided Fisher's exact tests [33] were used to determine the effects of purebred versus hybrid lineage (*n *= 13 and 12 families, respectively) and specific population cross (OO versus OW versus WW versus WO; electronic supplementary material, table S3) on larval survivorship (cultures scored as alive or dead). Linear models were used to assess the impacts of lineage purity (*n* = 23 larvae for each purebred and hybrid comparison) and specific population cross (electronic supplementary material, table S3) on larval weights. Models were run in the R base package ‘*Stats*’ [33], and the suitability of linear models was assessed with standard diagnostic plots. The package ‘*Multcomp*’ [34] was used to extract appropriate comparisons across the population crosses and perform post hoc Tukey's HSD tests, adjusted for multiple comparisons using the ‘single-step’ method. The impact of lineage purity and population cross on the number of settled individuals (*n *= 12 and 11 families for purebreds and hybrids, respectively; electronic supplementary material, table S3) and on the number of juveniles surviving in the field (*n *= 11 and 9 families for purebreds and hybrids, respectively; electronic supplementary material, table S3) was calculated using the non-parametric Kruskal–Wallis test [33]. Tukey's post hoc tests of significance were generated with generalized linear negative binomial models (glms) implemented in the package ‘*MASS*’ [35].

#### 2.5.2. Familial and parental effects on larval survival, weight and settlement success, and on juvenile mortality

The effects of parental identity on larval survivorship and larval weights were determined using the methods described above (electronic supplementary material, table S3). The familial and parental effects on settlement and the interactive effects of settlement number and parental identity on juvenile survival were assessed using glms and generalized linear mixed effects models (glmms; electronic supplementary material, table S3). A Poisson distribution with observations treated as a random effect was used to model how settlement varied by dam, sire and their interaction (all random effects). The same model was used to examine the effects of each dam and sire compared to dam/sire means by treating each in turn as a fixed effect. A negative binomial mixed model was used to assess how settlement abundance (fixed effect) and parental identity (dam, sire, interaction as random effects) influenced juvenile abundance (i.e. survival) in the field. The observation-level random effect in the first model and the negative binomial in the second model were used to account for overdispersion. The VarCorr function from the ‘*nlme*’ package [36] was used to assess the variability of the random effects using standard deviations. Glmms were run using the packages ‘*lme4*’ [37] and ‘*MASS*’ with Gauss–Hermite quadrature (GHQ). The addition of sire and sire-by-dam interaction accounted for no additional variability in the second glmm model and the model was refitted with dam-by-settlement interaction as a fixed effect. To determine the individual effect of each dam on juvenile survivorship and her interactive effect with settlement abundance, deviation coding (each level compared to the grand mean) was specified as above, with dam, settlement and their interaction as fixed effects. Settlement as a single term was not significant; it was dropped and the model was refitted with the interaction term and dam as fixed effects. The packages ‘*ggplot2*’ and ‘*effects*’ were used to visualize model predictions [38,39], and ‘*ggplot2*’ was also used to calculate the *R*^2^-value for comparison of larval weight and per cent mortality. *R*^2^-values were calculated in two ways to account for families that died at the larval culturing phase, with those families either counted as having 100% mortality or excluded from the analysis. Assumptions of homogeneity of variance, independence, nonlinearity, normality and overdispersion were assessed. Model fit was compared using AICc calculated from the R package ‘*MuMIn*’ [40]

### 2.6. *Symbiodinium* genotyping and analysis of field juveniles

Illumina sequencing of the ITS-2 locus was used to determine whether differences in *Symbiodinium* communities existed among the five coral families with the highest and three families with the lowest per cent mortality (table 1). All juveniles from the F_8_ family died, but six individuals could be sampled from the third (excluded) culture replicate that also had similarly high mortality (96%).

**Table 1.** Number of individually sequenced juveniles per coral family, for families with the highest and lowest per cent mortality, including the average number of mapped reads (post paired-end merging and filtering) across each family and their respective standard errors (s.e.), with the exception of F_17_, in which there was only one survivor.

family	mortality (%)	no. juveniles sequenced	average number mapped reads (±s.e.)
F_12_	0	12	77 999 ± 3468
F_4_	2.7	12	68 523 ± 5871
F_1_	6.9	13	56 262 ± 3930
F_14_	90.16	6	140 120 ± 15 198
F_28_	91.6	2	45 632 ± 28 825
F_18_	96.9	3	148 201 ± 35 468
F_17_	98.9	1	175 042 ± n.a.
F_8_	100	6	24 951 ± 11 404

DNA from single juveniles was extracted using Wayne's method [41] and lysed using a FastPrep-24 matrix 1 mm silicia spheres (MPBio) three times at 30 s and 4.0 m s^−1^. Library preparation and paired-end Miseq sequencing (Illumina) were performed at the University of Texas at Austin's Genomics Sequencing and Analysis Facility (USA), using the following primers: ITS2alg-F (5′-TCGTCGGCAGCGTCAGATGTGTATAAGAGACAGGTGAATTGCAGAACTCCGTG) and ITS2alg-R (3′-TTCGTATATTCATTCGCCTCCGACAGAGAATATGTGTAGAGGCTCGGGTGCTCTG-5′) [42]. A total of 55 samples were sequenced, with 1–13 juveniles per family (table 1). Raw reads were analysed using the USEARCH and UPARSE pipeline [43] (v. 7). Chimaeric reads were filtered and reads with an Expected Error greater than 1.0 were discarded [44]. Remaining reads clustered with the default 97% identity and minimum cluster size of 2 (thus eliminated all singleton reads), after which all reads were globally aligned to 99% similarity with gaps counted as nucleotide differences. After filtering and mapping, 1 848 018 reads remained (44.1% of raw reads), partitioned across 333 OTUs. OTUs were then annotated using BLAST+ run against the full non-redundant nucleotide NCBI database [45,46]. OTUs that did not blast to *Symbiodinium* accessions were removed manually (4.2%, many belonging to other Dinoflagellata) with 1 769 859 *Symbiodinium* specific reads belonging to 136 OTUs remaining. The majority of reads were attributed to clades A (36.2% reads, 16 OTUs), D (29.8%, 14), C (19.6%, 15), uncultured eukaryotes (10.3%, 45) and *Symbiodinium* identified from environmental sampling (environmental *Symbiodinium*) (2.6%, 31). Clades G (1.4%, 5), B (0.1%, 4), F (0.03%, 5) and H (0.007%, 1) were represented by far fewer reads and OTUs than those from C, D and A.

Cleaned sequence data were variance normalized using a geometric mean and shifted log transformation implemented in the ‘*DESeq2*’ package in R [47]. Differential abundance analysis was performed with the same package using the contrast function to extract specific comparisons from the negative binomial generalized linear model. OTUs that were found to have significantly different abundances and which were previously identified as either ‘uncultured eukaryotes’ or ‘environmental *Symbiodinium*’ using the full NCBI database were re-blasted to a custom-built database of all *Symbiodinium*-specific NCBI sequences. This allowed a reclassification of OTU19 to clade E (JN406302, [48]) and OTU4 to clade D. Non-metric multidimensional scaling (NMDS) was performed on variance-normalized OTU abundances using the packages ‘*Phyloseq*’, ‘*vegan*’ and ‘*ellipse*’ with a Bray–Curtis dissimilarity matrix [49–51]. NMDS analysis does not assume linear relationships between underlying variables and OTUs, and the resulting distances between samples calculated are indicative of their similarity, with samples positioned closer in space being more similar [52]. The use of a Bray–Curtis matrix allows for incorporation of the presence/absence data, as well as the abundance of OTUs. A permutational multivariate analysis of variance was used to determine whether *Symbiodinium* communities differed significantly between high and low mortality families using the ‘*adonis*’ function in ‘*vegan*’. The Chao1 metric describes species richness between families with differential mortality and was calculated in ‘*Phyloseq*’. This metric incorporates differences in library sizes between samples and is especially appropriate for datasets with many low abundance OTUs [53,54].

### 2.7. Multiple ITS-2 copies and intragenomic variation

*Symbiodinium* genomes are notoriously large and composed of multiple copies of intragenomic variants [55]. Intragenomic variation within and between *Symbiodinium* types makes classifying species-level diversity in *Symbiodinium* challenging [56,57]. Currently, diversity has only been definitively determined from simple communities of a few species using single-cell analyses [58] or approximated from serial dilutions [57,59]; diversity has not been verified for complex communities made up of hundreds of OTUs. To evaluate the presence of multiple copies and/or intragenomic ITS-2 variants, we employed an approach based on three criteria. Meeting all three of the following criteria was required to identify an OTU as being multi-copy and/or an intragenomic variant: (i) co-occurrence, (ii) proportionality and (iii) per cent similarity. Specifically, OTUs found to occur in regularly increasing or decreasing multiples of a baseline abundance (large *R*^2^-values) across a large number of samples might feasibly co-occur within the same cells and hence may be multiple copies of the same gene. A high per cent similarity is indicative of intragenomic variants having multiple copies of the same gene according to the above pattern [60], although multiple, divergent sequences may possibly coexist within single genomes simultaneously [61]. *Symbiodinium* types that are very divergent (low similarity) but occur in similar patterns across samples and in similar proportions may conversely represent divergent types that share similar ecological niches.

The following analyses were performed on the 10 OTUs that had significantly different abundances in high and low mortality families. OTUs were first separated by clade using initial BLAST+ identities (C: OTU1/OTU121/OTU162, environmental/clade E: OTU4/OTU19, *Symbiodinium*1635: OTU13/OTU124), and their co-occurrence across samples was identified using the tree function in ‘*Phyloseq*’, with the variance-normalized read depth described above. Correlations between these OTUs were calculated with the function ggpairs in the package ‘*GGally*’ [62]. Geometric distances and aligned pairwise similarity were calculated for these OTUs using the package ‘*ape’* [63] and Clustal Omega [64] with Geneious [65], respectively.

## 3. Results

### 3.1. Population effects on larval survival, weight and settlement, and on juvenile mortality

#### 3.1.1. Larval survival

Neither lineage purity (i.e. OO, WW versus OW, WO) nor population cross (i.e. OO versus WW versus OW versus WO) significantly affected the survival of larval cultures (Fisher's exact tests: *p* = 1 for both). This stage was survived by 92.3% of purebred lineages (12/13 purebred families had all three culture replicates survive) and 91.7% of hybrid lineages (11/12 hybrid families). All culture replicates persisted for all OO and OW families, and 83.3–85.7% of WO and WW crosses survived (5/6 and 6/7 families, respectively).

#### 3.1.2. Larval weights

**Figure 1.**
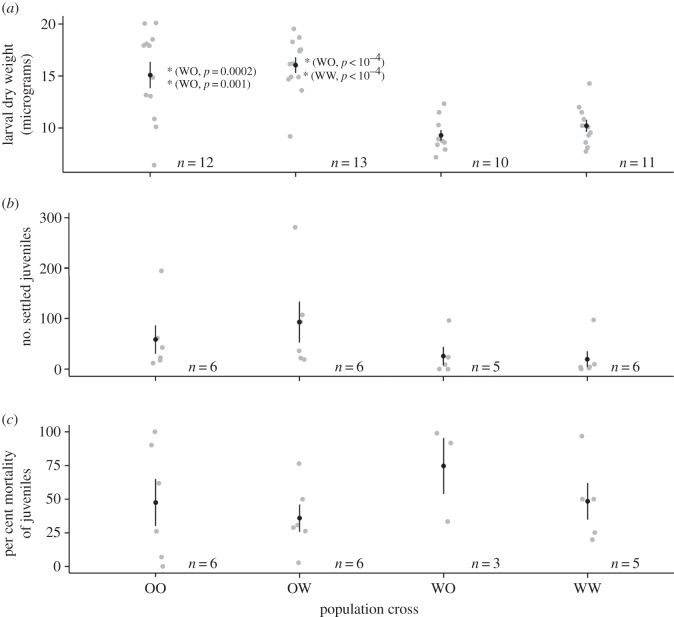
Variation among population crosses in (*a*) mean (±s.e.) dry weight of larvae; (*b*) mean (±s.e.) number of settled larvae and (*c*) mean (±s.e.) relative juvenile mortality (%) after 25 days of field exposure. Black circles are means; vertical bars are standard errors; light grey points are raw data; *p*-values indicate Tukey's post hoc significance test results of specific comparisons. For example, the mean larval dry weight of population cross OO was significantly greater (*p* = 0.0002) than the mean larval dry weight of cross WO. Sample sizes are indicated to the right of each population cross.

Larval weights did not differ significantly between purebred (OO, WW) and hybrid (OW, WO) larvae (linear model (LM): *F*_1,44 _= 0.084, *p* = 0.77), which, respectively, weighed 12.8 ± 0.9 and 13.1 ± 0.9 µg, on average. However, larval weights differed significantly among offspring of the four population crosses. Dry weights of larvae from purebred Orpheus lineages and Orpheus dam crosses were significantly heavier than weights of larvae from crosses involving Wilkie dams (15.1 ± 1.3–9.3 ± 0.5 µg, Tukey's post hoc test (TPT): *p *< 0.001, figure 1*a*). Larval weights did not differ significantly between OO and OW crosses (TPT: *p *= 0.85), nor between WO and WW crosses (TPT: *p *= 0.89; figure 1*a*).

#### 3.1.3. Settlement success

The number of settled juveniles did not vary with lineage purity (Kruskal–Wallis rank sum test (KW): $\chi_{1}^{2}=0.55$, *p *= 0.46), with purebred lineages having, on average, 39 ± 16.4 settled juveniles compared to 62 ± 24.8 settled juveniles in hybrid lineages. While crosses with Orpheus dams (OO, OW) had more settlers compared with crosses with Wilkie dams, these differences were not statistically significant (GLM, TPT: *p *= 0.17–0.99, figure 1*b*).

#### 3.1.4. Field mortality

Per cent mortality of juveniles after 25 days in the field was not significantly described by lineage purity (KW: $\chi_{1}^{2}=0.1,$ *p *= 0.73) or population cross (KW: $\chi_{3}^{2}=2.5,$ *p *= 0.47); 47.9 ± 10.8% and 48.8 ± 11% of purebreds and hybrids died, respectively. Juveniles of Orpheus dams crossed with Wilkie sires suffered the lowest mortality (35.9 ± 10.2%), while those from Wilkie dams crossed with Orpheus sires had the highest mortality (74.7% ± 20.8; figure 1*c*).

### 3.2. Familial and parental effects on larval survival, weight and settlement success, and on juvenile mortality

#### 3.2.1. Larval survival

Cultures for 92% of the families had larvae surviving at the end of the rearing stage (i.e. 23 of 25 families had all three culture replicates survive). Dam identity significantly affected larval survivorship (Fisher's exact test: *p *= 0.007), driven by the mortality of two families from Wilkie dam W7. Sire identity had no effect on larval survivorship (Fisher's exact test: *p* = 0.58). The two families that died were sired by O5 and W11.

#### 3.2.2. Larval weights

**Figure 2.**
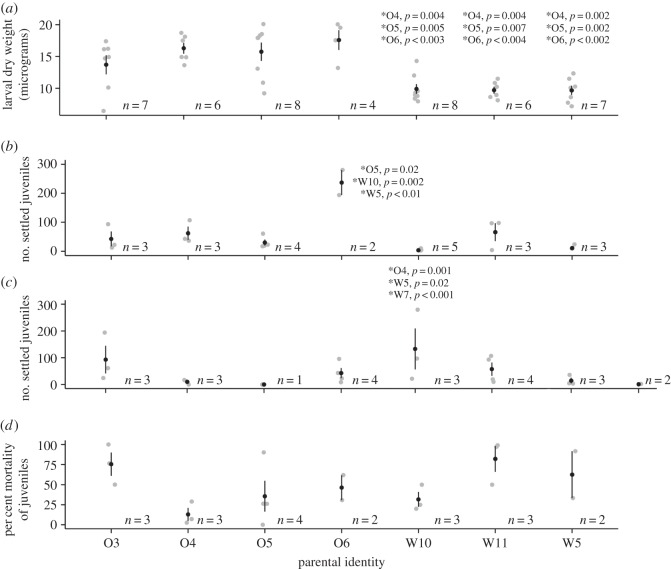
Variation among dams in (*a*) mean (±s.e.) dry weight (µg) of larvae per dam; (*b*) mean (±s.e.) number of settled larvae per dam; (*c*) mean (±s.e.) number of settled larvae per sire and (*d*) mean (±s.e.) relative mortality (%) of juveniles per dam during 25 days of field exposure. Black circles are means; vertical bars are standard errors; light grey points are raw data; *p*-values indicate Tukey's post hoc significance test results of specific comparisons. For example, the mean larval dry weight of parental identity W10 was significantly less (*p* = 0.004) than the mean larval dry weight of O4. Samples sizes are indicated to the right of each parental identity.

Mean larval weight varied significantly among dams (LM, TPT: *F*_6,39_ = 8.615, respective *p-values* below) and ranged from 17.6 ± 1.6 µg (dam O6) to 9.7 ± 0.73 µg (dam W5; figure 2*a*). Dam O6 larvae were significantly heavier (LM, TPT: *p *= 0.001–0.002) compared with larvae of all three Wilkie dams but not the other Orpheus dams (LM, TPT: *p *=* *0.35–1.0). The same pattern was true for dam O5 (LM, TPT: *p *= 0.004–0.007 and 0.8–1.0) and dam O4 (LM, TPT: *p *= 0.003–0.006, 0.7–1.0). The mean dry weight of dam O3 larvae did not differ statistically from mean larval weights of any other dam (LM, TPT: *p *= 0.15–0.81), with O3 larvae weighing less than those of the other Orpheus dams but more than mean larval weights of the Wilkie dams. The three Wilkie dams produced the lightest larvae, which did not differ significantly in weight from each other (LM, TPT: *p *= 1.0).

#### 3.2.3. Settlement success

Larvae from 86.9% of the families (i.e. 20 of 23 families) settled, with larvae of the remaining three families (F_21_, F_26_, F_30_) continuing to swim at day 19 pf, at which time larvae from the other families had settled and juveniles were deployed in the field. The GLMM analysis indicates that the random effects of dam, sire, their interaction and observation-level effect accounted for 61.9% of settlement variability. Of the 61.9%, maternal identity explained approximately 23.9% of the total variability in settlement, sire explained 37.9%, and the dam by sire interaction explained 9.91%. Dam O6 families had the largest number of settled juveniles, while dam W10 families had the lowest (figure 2*b*). Using dam O6 as a reference, the number of larvae settling differed significantly for dam O6 in comparison to dams O5, W10 and W5 (GLMM TPT: *p* = 0.002–0.02). Alternatively, with sire W10 as a reference, larvae from this sire settled significantly less in comparison to larvae from sires O4, W5 and W7 (GLMM TPT: *p* ≤ 0.001–0.02, figure 2*c*). The comparison in larval settlement between sires W10 and O5 was not significant, as larvae from only one family with sire O5 remained by this stage of the analysis (GLMM TPT: *p* = 1).

#### 3.2.4. Juvenile mortality on the reef

Substantial variation in juvenile mortality was detected among families, with an average mortality of 48.3 ± 7.5% after 25 days in the field; only a single family (F_8_) suffered 100% juvenile mortality. Mortality of juveniles ranged from 12.9 ± 8.1% for juveniles of Orpheus dams to 82 ± 16% for juveniles of Wilkie dams (figure 2*d*), with juveniles of dams from both locations suffering both high mortality (O3 and W11) and low mortality (O4 and W10). Initial juvenile abundance (i.e. settlement number), the combined random effects of dam/sire/dam × sire interaction, and residual variance accounted for 53.0%, 12.8% and 34.1% of the total variability in juvenile survivorship, respectively. Of the random effects, GLMM partitioned 37.9% of the variation (4.9% of the total) to maternal effects and 62.0% (7.9% of the total) was residual noise. There was no effect of sire or of dam × sire interaction on juvenile mortality. With the direct maternal effects on juvenile survival (4.9%) in addition to the carry-on indirect maternal effects on juvenile survival through settlement (23.9% of 53% = 12.6%), the total maternal contribution to juvenile survivorship was 17.5% (the sum of 4.9% and 12.6%).

**Figure 3.**
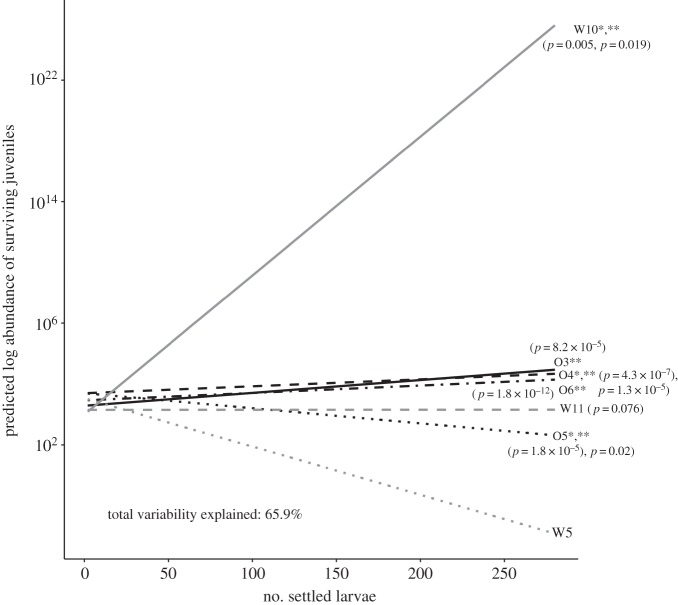
Relationship between predicted logged juvenile abundance after 25 days in the field, as explained by the number of settled larvae and dam identity based on outputs from the generalized linear model (GLM). Asterisks adjacent to dam labels signify significant effects due to dam identity and/or the dam × settlement interaction on juvenile survival derived from the generalized linear negative binomial model in which settlement abundance as a single factor has been dropped but remains as an interactive factor with dam identity. A single asterisk (*) signifies a significant effect due to dam identity, double asterisk (**) signifies a significant dam × settlement abundance effect. Note that the effect of dam W11 identity was only marginally significant (*p* = 0.076).

Once dam identity and the dam × settlement number interaction were included as fixed effects in the above model, the effect of larval settlement success on the abundance of surviving juveniles was no longer significant (GLMM TPT: *p *= 0.14), although both dam identity (O4, O5, W10, W11, GLMM TPT: *p *= 4.3 × 10^−7^–0.076) and the dam × settlement abundance interaction (O5, W10, GLMM TPT: *p *= 0.0097–0.015) were significant. We then refitted the model without settlement as a single factor (now only including dam and dam × settlement interaction). Modelled survival of juveniles from dams W10, O6 and O3 indicates that as settlement increases, predicted juvenile abundance increases, even with initially low settlement abundances (figure 3). Summary statistics indicate that this positive correlation may be predominantly due to dam identity alone in the case of dam W10 (GLMM TPT: *p *= 0.005) compared to the dam by settlement interaction (GLMM TPT: *p *= 0.019). Juveniles from dam W5 had very low survivorship regardless of settlement abundance, with no significant relationship between settlement number and dam identity on juvenile survival (GLMM TPT: *p *= 0.18). Survival of W11 juveniles showed marginal statistical evidence for an interaction with dam identity (GLMM TPT: *p *= 0.076), but modelled juvenile survival appeared to benefit little from increased settlement numbers (GLMM TPT: *p *= 0.99; figure 3). Poor survival of O5 juveniles was attributable to dam identity and dam by settlement interaction (GLMM TPT: *p *= 1.8 × 10^−5^, 0.02). The predicted abundance of juveniles of dams O3, O4 and O6 increased with an increase in settlement number due to the significant effect of dam by settlement interaction (GLMM TPT: *p *= 1.8^−12^–8.2 × 10^−5^), although predicted abundance of juveniles from dam O4 increased even at low settlement values, which indicates that the significance of the interaction may be predominantly from dam identity (GLMM TPT: *p *= 4.3^−7^). Finally, variation in juvenile survival was not well explained by larval weight for the seven dams that had surviving juveniles. This was true whether families that had failed settlement were included as 100% mortality (*R*^2^ = 0.328) or excluded completely (*R*^2^ = 0.201; electronic supplementary material, figure S1).

### 3.3. *Symbiodinium* community in families with high and low per cent mortality

**Figure 4.**
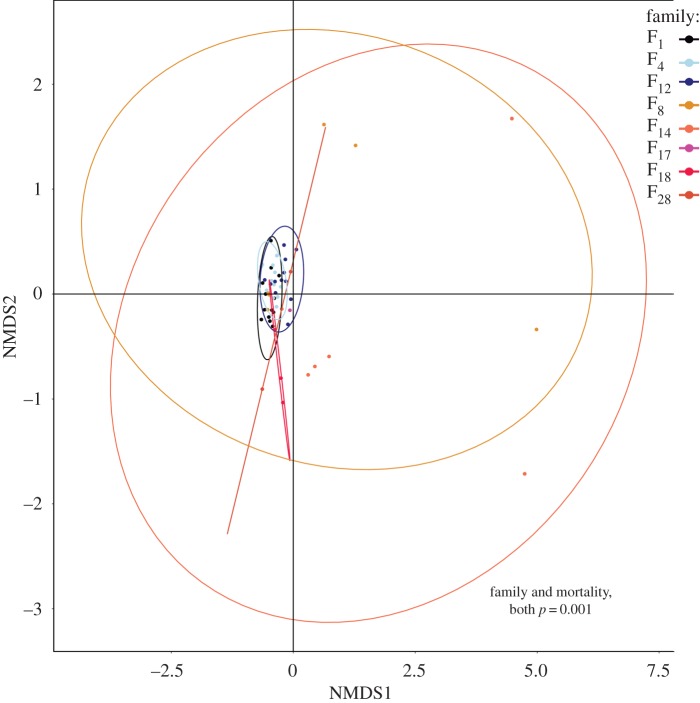
Non-metric multidimensional scaling (NMDS) plot using a Bray–Curtis distance matrix of variance normalized OTU abundances. Families with low mortality (less than 10%) are in cool colours and high mortality families (more than 90%) in warm colours. Each point represents a unique juvenile, where its family is indicated by colour. Ellipses with corresponding colours represent the 95% probability region for each family. Significant effects (*p* = 0.001) of family and mortality class (high versus low) are derived from permutational multivariate analysis of variance.

**Figure 5.**
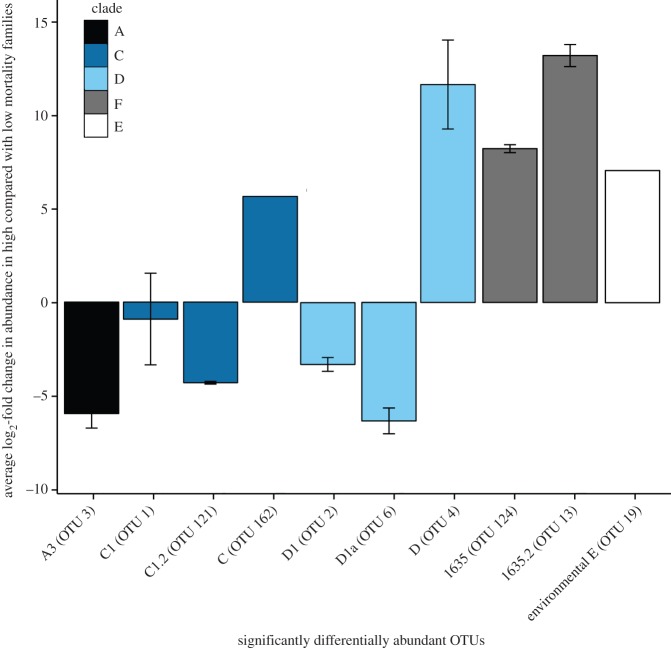
Average log_2_-fold changes in abundance in high (more than 90%) mortality families in comparison to low (less than 10%) mortality families. Log_2_-fold changes were calculated from negative binomial generalized linear models in DESeq2, with standard error bars representing significant Benjamini–Hochberg *p*-values (*p* < 0.05). Colours represent the classification of different *Symbiodinium* types in clades: A, C, D, F and environmental *Symbiodinium* (clade E).

Illumina sequencing of the ITS-2 locus revealed 136 *Symbiodinium*-specific OTUs across the 55 samples. *Symbiodinium* communities differed significantly in overall species richness and diversity between high and low mortality families (permutational multivariate analysis of variance; by family: *R*^2^ = 0.21, d.f. = 6, *p* = 0.001; by mortality: *R*^2^ = 0.18, d.f. = 1, *p* = 0.001). High mortality families had a greater number of *Symbiodinium* types present (average Chao1 ± s.e.: 16.4 ± 1.7) compared to low mortality families (12.7 ± 2.7 *Symbiodinium* types). Variability in OTU richness and abundance was greater within families of high mortality crosses (between juveniles of the same families) than within low mortality families (Bray–Curtis, NMDS; figure 4). Juveniles from high mortality families differed markedly from each other, with *Symbiodinium* communities of some individuals resembling the conservative diversity of low mortality families while others were more variable. *Symbiodinium* communities associated with juveniles of high mortality families had significantly lower abundance of A3, D1, D1a and two C1 *Symbiodinium* types, and greater abundance of two F and one C type, and of a clade E environmental *Symbiodinium* type compared with juveniles of low mortality families (figure 5, GLM Benjamini–Hochberg (BH): *p*_adj_ = 6.15 × 10^−28^  − 0.039; electronic supplementary material, table S2).

*Symbiodinium* communities associated with juveniles of the three low mortality families were similar in both *Symbiodinium* OTU richness and abundance (Bray–Curtis, NMDS, figure 5); however, the abundances of some *Symbiodinium* types differed significantly among low mortality families. Family 1 juveniles had 3.1-fold less D1a (BH *p*_adj_ = 0.001) and 3.7-fold more C1 (BH *p*_adj _= 0.0009) compared with F_4_ juveniles, but the abundances of all *Symbiodinium* types were similar compared with abundances of *Symbiodinium* types associated with juveniles of Family 12. Concomitantly, *Symbiodinium* communities associated with F_12_ juveniles differed from those of F_4_ juveniles, with 3.6-fold less C1 (BH *p*_adj_ = 0.0007) and 3.3-fold more D1a (BH *p*_adj_ = 0.0007).

#### 3.3.1. Intragenomic variants

We did not detect evidence of intragenomic/multi-copy variants in the 10 OTUs that distinguished high and low mortality families based on our three criteria. There was not a strong level of co-occurrence in any of the OTUs. Within clade C, OTU1 and OTU121 co-occurred in 42 of the 65 samples, but neither co-occurred with OTU162. OTU13 was present in four additional samples compared with OTU124. Similarly, OTU4 was found in 11 additional samples compared with OTU19. Where OTUs did co-occur, only OTU13 and OTU124 had a strong level of proportionality (*R*^2 ^= 0.992), OTU1 and OTU121 had moderate proportionality (*R*^2 ^= 0.739), and all other combinations of OTUs had very low proportionality (*R*^2 ^= −0.0551–0.06). Pairwise geometric distance analysis indicated that OTU1 and OTU121 had 57.6% pairwise similarity, whereas aligned pairwise per cent similarity analysis indicated 96.2% pairwise similarity. OTU13 and OTU124 had 76.4% and 96.9% pairwise similarity, respectively, based on these two analyses. Both comparisons were characterized by multiple base pair differences and blocks of deletions.

## 4. Discussion

### 4.1. Maternal influence on density-dependent and -independent juvenile mortalities

Here, we present evidence of maternal effects on the survival of early life-history stages of the coral *Acropora tenuis* during the first month of development on the reef*.* Maternal identity underpinned 23.9% of the variability found in larval settlement success and explained 17.5% of variability in juvenile survival. By contrast, neither paternal effects nor paternal by maternal interaction significantly impacted juvenile survivorship in the field. Our results on larval settlement and juvenile survival indicate that maternal carry-on effects reach through several developmental stages in a broadcast-spawning coral. Importantly, it appears that the initial number of settled juveniles is not a significant factor in predicting the number of survivors once maternal identity is taken into account. Initial settlement number was only significant when its interaction with certain dam identities was included, creating conditions of both density-dependent and -independent mortalities driven by dam identity. Initial settlement numbers were not important for dams W10 and W5, with all juveniles either surviving or dying, respectively, regardless of initial settlement numbers. This pattern was especially clear for dam W10, which produced families with very low numbers of settlers that still resulted in very high juvenile per cent survival (F_22_, F_23_ and F_24_). It is unlikely that families with high mortality died due to genetic incompatibility between (i.e. selfing between clonemates), given patterns of both very high and very low survivorship found for families resulting from crosses involving colonies from the same population (e.g. O3 × O4 and O3 × O5 failed, but O4 × O5 was successful).

These findings suggest that some aspect(s) of maternal identity, either at an environmental or genetic level, directly influences the fitness of early life-history stages, predisposing juveniles to either live or die. At an ecological level, this signifies that juvenile survivorship is not merely a product of fecundity of the parent colony or initial recruitment numbers, and that high recruitment does not guarantee greater survival and community representation. The maternal effects on larval settlement and juvenile survival demonstrated here provide evidence that differences in colony identity may scale up through differential survival of early life-history stages and contribute significantly to shaping heterogeneous reef communities. Maternal identity should be taken into consideration as an important factor contributing to juvenile survival and population demography, in conjunction with currently recognized factors like predation, competition and disease.

### 4.2. Impacts of *Symbiodinium* community on survival of coral juveniles

The *Symbiodinium* community acquired by juveniles of broadcast-spawning corals offers a potential link between maternal effects and juvenile survivorship. Juvenile families had different symbiont communities after less than one month of field deployment and exposure to *Symbiodinium*, representing the earliest record of symbiont specificity in a coral species with horizontal transmission yet reported, potentially due to the deep sequencing approached used. Microbes (both bacterial and eukaryotic) are known to heavily impact coral development and health [66–69], but few studies to date investigate the links between parental identity (maternal or paternal effects) and *Symbiodinium* assemblage acquired by juveniles with horizontal symbiont transmission. Our results identify a potential link between maternal identity and the juvenile *Symbiodinium* community that may have an environmental or genetic basis, and propose that an inherited or transmitted mechanism, whose expression differs across dams, leads to differential formation of *Symbiodinium* communities among families and contributes to differences in juvenile survival. Specifically, juveniles of low mortality families had a more conserved *Symbiodinium* community compared with high mortality families, consistent with greater selectivity for specific *Symbiodinium* types. Low mortality families also contained a high proportional abundance of *Symbiodinium* types A3 and D1, suggesting that these are critical symbionts for survival of *Acropora tenuis* juveniles. Increased survivorship of *Acropora yongei* juveniles in Japan was also found to be associated with a significantly greater abundance of clade A *Symbiodinium* [16]. While the authors attributed high survivorship specifically to the early acquisition of symbionts at the larval stage, they acknowledge that the selective uptake of specific symbiont types could have led to differential survivorship. Our data support this latter conclusion and highlight that the acquisition of *Symbiodinium* A3 is ecologically essential to early juvenile survivorship in species of *Acropora*. Alternatively, differences among the *Symbiodinium* communities detected here may be the result, rather than the driver, of mortality, with some unmeasured factor compromising host–symbiont regulation, leading to dysbiosis and death. Further work is needed to elucidate these alternative explanations, as well as the fitness benefits and costs of hosting particular symbiont communities.

It is currently unclear whether *Symbiodinium* uptake in early life stages of corals is specific [16] or random [70]; however, our results suggest that the high mortality experienced by certain juvenile families may be due to either the specific absence of A3 or to the non-specific uptake of a community that becomes detrimental for the juvenile coral host [71]. According to this latter interpretation, it is not the presence or the absence of single symbionts that cause a decrease in host fitness, but the community composition and their interactions with a specific host genotype and environment that are detrimental. Non-selective community uptake may influence juvenile survival in two ways: (i) it may expose juveniles to opportunistic or suboptimal *Symbiodinium* types and (ii) while the additional *Symbiodinium* types may not be opportunistic, they may occupy niche space and influence the density of other more beneficial *Symbiodinium* types. Both of these signatures can be seen in our data. We found that more diverse symbiont communities were generally associated with high mortality families and that those juveniles had sevenfold higher proportions of an environmental, potentially novel opportunistic *Symbiodinium* type (clade E OTU19). This type was only found in 1 out of 37 low mortality juveniles versus 9 out of 28 high mortality juveniles. Secondly, the main OTUs from the low mortality families (OTUs 1,2,3,6,121) were also at lower abundances in high mortality juveniles, with a concomitant fold-increase in certain OTUs (4,13,19,124,164). A similar increase in the abundance of non-beneficial fungi was documented in *Hydra* in the absence of the specific microbial community typically associated with healthy hosts, causing mortality in those individuals infected [72,73].

Differences in the variability of *Symbiodinium* assemblages in our study highlight the role coral-associated microbes can play in shaping the abundance and diversity of reef communities at local spatial scales through differential impacts on coral juvenile growth and mortality. Further support for this hypothesis is provided by microbial-driven density-dependent mortality of juveniles through increased pathogen transmission when juvenile densities are high [74,75], as well as mortality of adults in zones of competitive exclusion with turf algae, which cause hypoxic zones next to coral tissue and subsequent increases in virulent bacteria [76]. Also, the early acquisition of *Symbiodinium* increases juvenile survivorship and growth [16,23]. Evidence that maternal effects influence juvenile survival, combined with the fact that low mortality families had conserved *Symbiodinium* assemblages, provide a potential link between maternal identity and the composition of the *Symbiodinium* communities associated with juvenile corals, with some dams able to provide their offspring with mechanisms to select for beneficial communities (or exclude non-beneficial ones) while others do not.

The maternal effects detected here may have several underlying mechanisms that can be environmental and genetic [77]. Maternal environmental effects may be caused by differences in energy provision (lipid) provided to larvae, variations in egg or sperm quality due to colony age, or through epigenetic effects (reviewed in [15,78]). Another potential mechanism is genetic maternal effects through heritability of maternal genes that influence the development of an appropriate immune system in coral juveniles. Cell-to-cell recognition and phagocytosis have been hypothesized as key precursors to the establishment and maintenance of symbiosis with *Symbiodinium* [79] and may underpin maternal genetic effects through the transmission of genes that regulate a repertoire of immune-related, cell-surface proteins adapted for certain environmental conditions and/or symbiont types. A third possibility is that maternal identity affects juvenile survival through an unmeasured mechanism that results in dysbiosis of the symbiont community and juvenile mortality, as discussed above. These findings demonstrate that further research is needed to identify mechanisms underlying these maternal and familial effects in juveniles.

### 4.3. No evidence of local adaptation in northern and central populations of *Acropora tenuis*

We found that variation in larval survival, larval settlement success and field survivorship of juveniles was greater among families than between the two populations. While definitive statements about local adaptation of the northern and central GBR populations are not possible because juveniles were not reciprocally transplanted between the two locations, we present preliminary evidence for the lack of a significant effect of either lineage purity or population cross on per cent mortality of juveniles. Our results suggest that there was no fitness benefit associated with outplanting on their natal reef for Orpheus juveniles (either OO or OW), and no fitness cost associated with a non-natal grow-out location for Wilkie juveniles (either WW or WO). The lack of a strong population effect in our data suggests a lack of local adaptation, which is surprising given previous evidence of population genetic structure in corals [80,81] and the benefits of being locally adapted to the parental environment [82]. However, the absence of locally adapted populations within species' ranges can occur when reproductive propagules disperse widely, or if environmental variation is low and at a scale smaller than propagule dispersal [82]. This would be expected for many marine broadcasting species due to the high dispersal potential of currents. Indeed, the population genetic structure of *Acropora* species appears to be open in the Northern and Central sections of the GBR [83], presumably because the longevity of *A. tenuis* propagules (up to 69 days) would enable them to disperse far from maternal colonies [84]. Therefore, larval dispersal may be sufficiently high between Wilkie and Orpheus populations for gene flow to lead to fairly well-mixed populations. A similar study of *Porites astreoides* also concluded that population origin had little to no impact on juvenile survival [6]. Interestingly, southern GBR juveniles appear to be locally adapted [85], a finding in line with microsatellite data showing that the southern GBR represents a distinct region according to population structure analyses [83]. Finally, the lack of a population effect may also be due to the directionality of the outplanting environment. While other environmental factors may be equally or more important than temperature (e.g. turbidity, nutrients and currents), annual temperatures at Orpheus, where the juveniles were raised, are lower than those at Wilkie [4]; therefore, all juveniles were exposed to temperatures within their normal thermal ranges. In a concurrent study of the impact of heat stress on larvae from the two populations, Orpheus juveniles only exhibited lower survival when they were subjected to increased or higher temperatures found at Wilkie latitudes [4].

### 4.4. Differing reproductive modes and symbiont transmission strategies may drive the magnitude of parental effects on coral fitness

Reproductive mode is commonly assumed to be an important factor when assessing juvenile mortality risk [86], but empirical comparisons of maternal influences on the survival of juveniles originating from broadcast versus brooding modes are lacking. Unlike *A. tenuis*, brooding coral species have internal fertilization and gestation of planula larvae which are provisioned with *Symbiodinium* through vertical transmission. One could hypothesize that the magnitude of parental or maternal effects should be greater in brooding versus broadcasting corals. For example, brooded larvae (i) are exposed to the maternal environment, (ii) are typically larger than larvae developed from spawned gametes, (iii) acquire maternally derived symbionts and (iv) are almost immediately competent to settle, leading to typically shorter dispersal distances from maternal colonies. Consistent with this line of reasoning, 94% of variation in juvenile survival has been attributed to parental genetics in the brooding coral, *Porites astreoides*, compared to the estimated 17.5% in our study, although both species achieved similar mortality, on average, within the first months (61% versus 51.7% survivorship, respectively) [6]. These results suggest that the magnitude of parental effects may be tied to modes of reproduction and symbiont transmission, highlighting crucial but understudied links between parental identity, *Symbiodinium* community composition and local adaptation.

## 5. Conclusion

We show that maternal identity impacts multiple early life stages of the broadcast-spawning coral *Acropora tenuis*. Maternal identity is particularly important in determining juvenile survivorship, but initial settlement abundance was typically de-coupled, although this varied by colony. We also show that juveniles of low mortality families have a distinct and conserved *Symbiodinium* community, including a *Symbiodinium* type (A3) that is potentially crucial for early juveniles, and propose that maternal identity and juvenile survivorship are linked through the transmission of environmental and/or genetic mechanisms that influence symbiont community regulation. Future studies assessing the drivers of juvenile mortality and coral reef restoration efforts should consider maternal identity and *Symbiodinium* community composition of juveniles, along with other well-known abiotic and biotic mechanisms, as being important in shaping reef landscapes.

## Supplementary Material

2. Supp.Fig.1.weights_surv This TIFF is supplementary figure 1.

## Supplementary Material

1. Supplementary Material KMQ_final: This word file includes 3 tables and the figure legend for supplementary figure 1.

## Data accessibility

All raw sequencing data have been deposited in the NCBI Sequence Read Archive under Accession number SRP077416 and raw data files and statistical analysis can be found in Dryad at http://datadryad.org/resource/doi:10.5061/dryad.8b5g6 [87].
