# Supplementary figures and images for: Maternal effects and Symbiodinium community composition drive differential patterns in juvenile survival in the coral Acropora tenuis
Source: R Soc Open Sci. 2016 Oct 19;3(10):160471. doi: 10.1098/rsos.160471 (PMC5098987; doi:10.1098/rsos.160471)

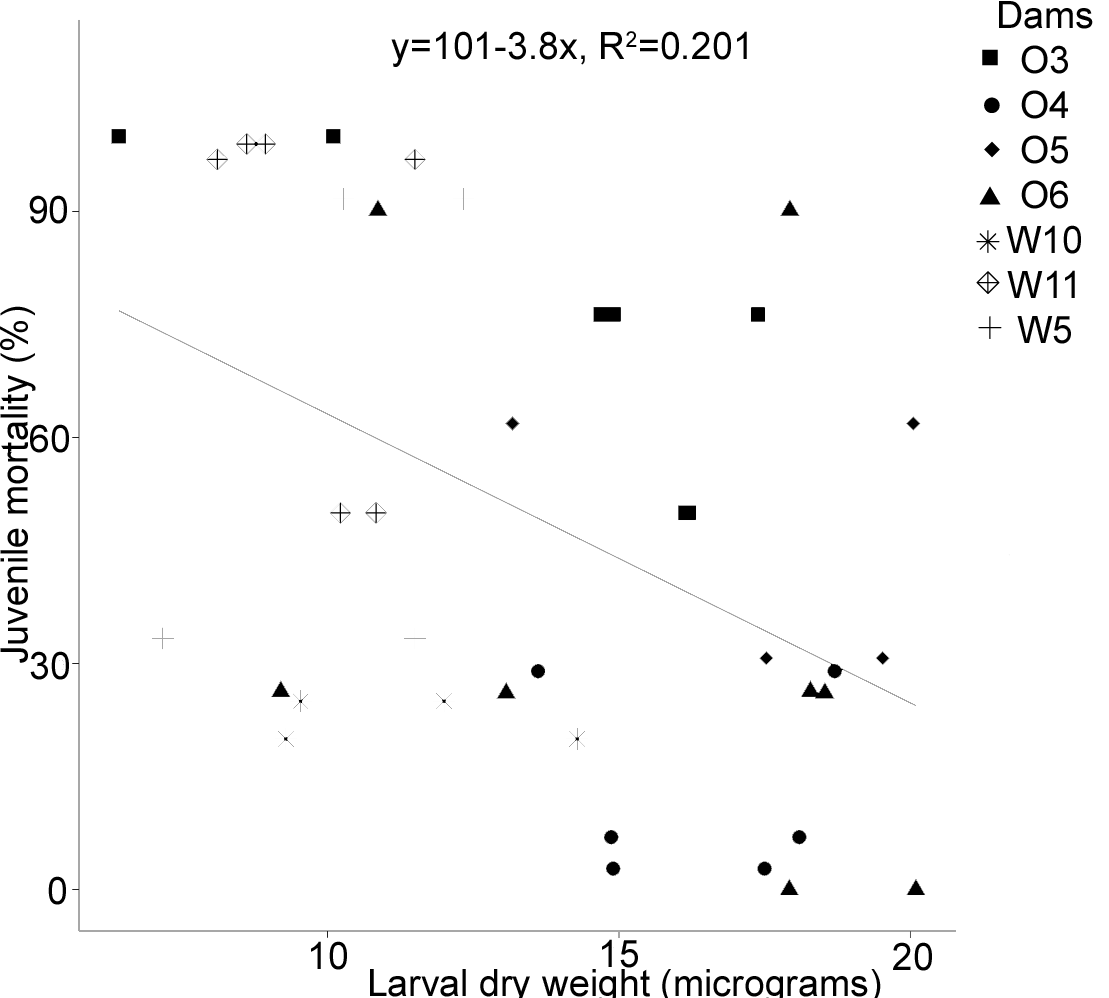

Supplement: 2. Supp.Fig.1.weights_surv This TIFF is supplementary figure 1. [file rsos160471supp1.tif]
