# Supplementary material for: Maternal effects and Symbiodinium community composition drive differential patterns in juvenile survival in the coral Acropora tenuis
Source: R Soc Open Sci. 2016 Oct 19;3(10):160471. doi: 10.1098/rsos.160471 (PMC5098987; doi:10.1098/rsos.160471)
Supplement: 1. Supplementary Material KMQ_final: This word file includes 3 tables and the figure legend for supplementary figure 1. [file rsos160471supp2.docx]

**Supplementary material**

Supplementary Figure. 1. Variation in percent mortality explained by variation across larval dry weights (micrograms). Each point represents a unique larva and shapes represent dam identity.

Supplementary Table 1. Summary of the 25 reproductive crosses created.

| Dam | Sire | | | | | | | | |
| --- | --- | --- | --- | --- | --- | --- | --- | --- | --- |
|  |  | O4 | O6 | O3 | O5 | W11 | W10 | W7 | W5 |
|  | O4 |  | F1 |  |  | F2 |  |  | F4 |
|  | O6 |  |  | F5 |  |  | F6 |  |  |
|  | O3 | F8 |  |  |  | F9 | F10 |  |  |
|  | O5 | F12 | F13 | F14 |  | F15 |  |  |  |
|  | W11 |  | F17 |  |  |  | F18 |  | F19 |
|  | W10 | F26 |  |  | F21 | F22 |  | F23 | F24 |
|  | W7 |  |  |  | F25 | F29 |  |  |  |
|  | W5 |  | F27 | F28 |  |  |  | F30 |  |

Supplementary Table 2. Log_2_fold change and corresponding adjusted p-value for comparisons of juveniles from different families. Families listed horizontally correspond to high surviving families and those listed vertically are low surviving families.

|  | F1 | | | F4 | | | F12 | | |
| --- | --- | --- | --- | --- | --- | --- | --- | --- | --- |
|  | Type | Logf2FoldChange | Padj | Type | Logf2FoldChange | Padj | Type | Logf2FoldChange | Padj |
| F8 | OTU_13 1635 | 12.799 | 1.46E-08 | OTU_13 1635 | 12.43216 | 4.27E-07 | OTU_13 1635 | 14.35262 | 3.61E-10 |
|  | OTU_3 A3 | -3.58571 | 1.31E-03 | OTU_6 D1a | -5.176843 | 1.24E-05 | OTU_3 A3 | -4.771737 | 4.11E-06 |
|  | OTU_1 C1 | -3.39679 | 5.44E-03 | OTU_2 D1 | -4.174351 | 4.44E-05 | OTU_124 163 | 8.455486 | 2.26E-03 |
|  | OTU_124 1635 | 7.80379 | 6.59E-03 | OTU_3 A3 | -4.039664 | 6.43E-04 | OTU_1 C1 | -3.309038 | 3.82E-03 |
|  | OTU_2 D1 | -2.14304 | 2.84E-02 | OTU_124 1635 | 8.421941 | 1.30E-02 | OTU_2 D1 | -2.173176 | 2.08E-02 |
|  | OTU_121 type | -4.37999 | 2.84E-02 |  |  |  | OTU_121 type | -4.228729 | 2.33E-02 |
| F14 | OTU_4 Uncultured | 17.50639 | 1.35E-29 | OTU_4 Uncultured | 16.15093 | 7.54E-27 | OTU_4 Uncultured | 17.11659 | 6.15E-28 |
|  | OTU_6 D1a | -5.24123 | 4.10E-07 | OTU_6 D1a | -8.386725 | 6.36E-17 | OTU_3 A3 | -5.394703 | 1.04E-07 |
|  | OTU_3 A3 | -4.208676 | 5.49E-05 | OTU_2 D1 | -4.643967 | 4.53E-07 | OTU_6 D1a | -5.020632 | 7.22E-07 |
|  | OTU_2 D1 | -2.612665 | 8.72E-03 | OTU_3 A3 | -4.66263 | 5.65E-06 | OTU_2 D1 | -2.642792 | 4.71E-03 |
|  |  |  |  | OTU_1 C1 | 3.986333 | 4.94E-04 |  |  |  |
| F17 | NA |  |  | OTU_2 D1 | -4.488758 | 0.0393881 | NA |  |  |
|  | NA |  |  | OTU_6 D1a | -4.976673 | 0.0393881 | NA |  |  |
|  | NA |  |  | OTU_162 C | 5.644083 | 0.0415672 | NA |  |  |
| F18 | OTU_3 A3 | -8.409899 | 2.22E-10 | OTU_3 A3 | -8.863853 | 2.38E-12 | OTU_3 A3 | -9.595925 | 2.07E-13 |
|  | OTU_4 Uncultured | 6.953951 | 2.60E-03 | OTU_2 D1 | -3.495286 | 3.54E-03 | OTU_ 4 Uncultured | 6.56415 | 7.33E-03 |
|  |  |  |  | OTU_4 Uncultured | 5.598488 | 3.54E-03 |  |  |  |
|  |  |  |  | OTU_6 D1a | -3.704052 | 4.05E-03 |  |  |  |
| F28 | OTU_6 D1a | -7.166145 | 5.82E-05 | OTU_6 D1a | -10.31164 | 1.58E-11 | OTU_6 D1a | -6.945547 | 0.00014167 |
|  |  |  |  | OTU_19 Uncultured | 7.073431 | 9.88E-03 |  |  |  |

Supplementary Table 3. Sample sizes for analyses: 1) population hybrids (OO, WW vs. OW, WO); 2) population cross (OO vs. WW vs. OW vs. WO); 3) maternal or paternal identity (dam, sire) for each of the treatment comparison (larval survivorship and weight, settlement and juvenile survivorship).

| Population cross | Larval survivorship (n=) | | Larval weights  (n=) | | Settlement  (n=) | | Survivorship  (n=) | |
| --- | --- | --- | --- | --- | --- | --- | --- | --- |
| OO | 6 | | 12 | | 6 | | 6 | |
| OW | 6 | | 13 | | 6 | | 6 | |
| WO | 6 | | 10 | | 5 | | 3 | |
| WW | 7 | | 11 | | 6 | | 5 | |
|  |  | |  | |  | |  | |
|  | Dams | Sires | Dams | Sires | Dams | Sires | Dams | Sires |
| O3 | 3 | 3 | 7 | NA | 3 | 3 | 3 | 3 |
| O4 | 3 | 3 | 6 | NA | 3 | 3 | 3 | 2 |
| O5 | 4 | 2 | 8 | NA | 4 | 1 | 4 | 0 |
| O6 | 2 | 4 | 4 | NA | 2 | 4 | 2 | 4 |
| W5 | 3 | 3 | 7 | NA | 3 | 3 | 2 | 3 |
| W7 | 2 | 2 | NA | NA | NA | 2 | NA | 1 |
| W10 | 5 | 3 | 8 | NA | 5 | 3 | 3 | 3 |
| W11 | 3 | 5 | 6 | NA | 3 | 4 | 3 | 4 |
